# M-Mode Echocardiography in Canine Veterinary Practice: A Comprehensive Review of Left Ventricular Measurements in 44 Different Dog Breeds

**DOI:** 10.3390/ani13182986

**Published:** 2023-09-21

**Authors:** Maria Cerbu, Constantin Cerbu, Ionel Papuc

**Affiliations:** 1 Department of Comparative Anatomy, Faculty of Veterinary Medicine, University of Agricultural Sciences and Veterinary Medicine, 400372 Cluj-Napoca, Romania; maria.vilcu@usamvcluj.ro (M.C.); ionel.papuc@usamvcluj.ro (I.P.); 2 Department of Infectious Diseases, Faculty of Veterinary Medicine, University of Agricultural Sciences and Veterinary Medicine, 400372 Cluj-Napoca, Romania

**Keywords:** veterinary cardiology, dogs, breed, echocardiography, M-mode

## Abstract

**Simple Summary:**

This review article focuses on canine M-mode (motion mode), particularly for assessing the left ventricle measurements in several dog breeds. It traces the evolution of echocardiography techniques, highlighting A-mode, B-mode, and M-mode for accurate unidimensional cardiac structure records. This article emphasizes M-mode’s significance in diagnosing conditions like MMVD, where identifying cardiac enlargement requires measuring left ventricular end-diastolic internal diameter corrected with body weight (LVIDdN). Also, M-mode’s role in DCM diagnosis is explained, noting criteria such as left ventricular dilatation. This review compiles data from various scientific sources to establish a methodology, presenting a detailed table of M-mode measurements for different breeds, ages, and sexes. In essence, our review underscores M-mode echocardiography’s crucial role in diagnosing and managing cardiac diseases in dogs. It highlights the significance of breed-specific reference values and offers a comprehensive summary of such measurements for diverse dog breeds, benefiting both clinicians and researchers.

**Abstract:**

This review article focuses on the use of canine M-mode in veterinary medicine, specifically in assessing the left ventricle measurements in several breeds. It traces the historical development of echocardiography techniques, including A-mode, B-mode, and motion mode (M-mode), which provide accurate unidimensional records of cardiac structures. This article highlights the significance of M-mode measurements in diagnosing stage B2 of MMVD, where left ventricular end-diastolic internal diameter corrected with body weight (LVIDdN) is essential for identifying cardiac enlargement. It also explains the role of M-mode in diagnosing DCM, outlining criteria such as left ventricular dilatation. The authors emphasize the importance of breed-specific reference values for echocardiographic measurements due to variations in somatotype among dogs. This review provides a comprehensive table summarizing M-mode measurements of the left ventricle for 44 different dog breeds, including interventricular septum thickness, left ventricular internal diameter, and left ventricular posterior wall thickness during systole and diastole. This review’s methodology involves compiling data from various scientific literature sources, providing an extensive tabular representation of M-mode measurements for different breeds, ages, and sexes. Overall, this review highlights the critical role of M-mode echocardiography in diagnosing and managing cardiac diseases in dogs, underscores the importance of breed-specific reference values, and presents a comprehensive summary of M-mode measurements for various dog breeds, aiding both clinicians and researchers.

## 1. Introduction

Transthoracic echocardiography (TTE) has been routinely used in veterinary medicine since the early 1980s to assess the dynamic morphology and function of the canine heart [1,2]. A-mode (amplitude mode) echocardiography was first used where reflected echoes were displayed as peaks on an oscilloscopic monitor [3]. This allowed the identification of cardiac structures based on peak distribution in time, amplitude, and intensity. B-mode (brightness mode) was later developed, representing reflected signals as fixed dots with brightness proportional to signal intensity. The addition of a time scale to the B-mode led to the development of the motion mode (M-mode) technique, displaying reflected echoes as vertical lines side by side on a time axis, allowing analysis of structures crossed by the ultrasonic beam [3]. Therefore, the echocardiographic M-mode provides a highly accurate unidimensional record of structures crossed by the ultrasonic beam.

Cardiac measurements are commonly determined using either the M-mode or the two-dimensional (2D) echocardiography, both of which allow measurement of left ventricular (LV) dimensions [1]. It is primarily used in cardiology to measure interventricular septum thickness (IVS), left ventricular internal dimensions (LVD), and left ventricular posterior wall thickness (LVPW) during systole and diastole. It can be interpreted from two views: 1. the right parasternal long-axis at the mitral valve level; and 2. the right parasternal short-axis view at the chordae tendineae level [4,5]. Schober and Baade (2000) found that M-mode measurements varied depending on the section approached, with long-axis sections showing slightly higher values compared with short-axis sections (around 5% difference) in healthy dogs. However, in dogs with cardiac pathology, these differences were statistically relevant, particularly for the diameter of the left ventricle in systole and diastole and the size of the interventricular septum in systole. Therefore, it is recommended to avoid interchanging sections, especially in patients with cardiac diseases [6]. Even if M-mode echocardiography is the most commonly used method for measuring left ventricular (LV) dimensions, it has several limitations: it is one-dimensional and it relies on geometric assumptions that may not hold true in all disease states [7].

Various attempts have been made to establish better correlations between M-mode measurements and body weight. Logarithmic or second-order polynomial models were found to predict reference values for M-mode measurements of cardiac chamber size more accurately than simple linear models. However, for M-mode measurements of cardiac wall thickness, logarithmic and polynomial models did not outperform simple linear models [8]. Since 2004, allometric scaling has been used and appears to be applicable to normal adult dogs of most breeds. Mean values and prediction intervals were calculated for normal dogs, allowing veterinarians to correctly index M-mode values. A commonly used formula for normalization is LVIDdN = LVIDd (cm)/weight (kg)^0.294^ [9]. An updated equation proposes the following: LVIDdN = LVIDd (cm)/weight (kg)^0.299^ [10]. It is worth noting that the updated equation is not yet recognized by the American College of Veterinary Internal Medicine (ACVIM) or the European College of Veterinary Internal Medicine (ECVIM).

The most common heart diseases in dogs are myxomatous mitral valve disease (MMVD) and idiopathic dilated cardiomyopathy (DCM) [11,12,13]. Diagnosis for both conditions relies on measurements obtained from M-mode echocardiography. For the diagnosis of Myxomatous mitral valve disease, one of the four criteria that identify stage ACVIM B2 in dogs is an increase in the left ventricular chamber size [11]. To assess the severity of cardiac enlargement, measurement of left ventricular end-diastolic internal diameter corrected with body weight (LVIDdN) is necessary [9]. The criterion for identifying advanced stage B2 of MMDV in dogs is an LVIDdN value of ≥1.7 [11,14]. The combined use of standard 2D and M-mode echocardiography along with conventional Doppler examination plays a critical role in assessing dogs affected by MMDV [15].

The European Society of Veterinary Cardiology (ESVC) task force provides guidelines for diagnosing DCM primarily based on M-mode and 2D echocardiography. Diagnosis of DCM requires the presence of the following three criteria: (i) left ventricular dilatation, (ii) reduced systolic function, and (iii) increased sphericity of the left ventricle [13]. Hence, M-mode in canine echocardiography is an indispensable diagnostic tool. Initially, M-mode measurements were taken from the trailing endocardial edge of the anterior wall to the leading endocardial edge of the posterior wall of each observed structure. Nowadays, the leading edge method [16] is recommended, where measurements are taken from the leading endocardial edge of the anterior wall to the leading endocardial edge of the posterior wall of the structure of interest [17].

Advancements in the diagnosis and monitoring of cardiovascular diseases in dogs are continuously being made. Various variables, such as breed, somatotype, sex, age, body weight (BW), heart rate (HR), and athletic condition, have been reported to impact cardiac measurements [18,19,20]. Commonly used reference ranges in canine cardiology come from populations of multiple breeds and centers, and they depend on BW [1,9,10,19,21]. Breed-specific reference values are necessary to more accurately evaluate the heart due to the significant variation in somatotype among dogs. Currently, there are about 40 breed-specific echocardiographic studies, with several breeds showing significant differences compared with the general population of healthy mixed-breed dogs.

Generic reference values for echocardiographic measurements have been published for a wide dog population [1,9,17,18,21,22,23,24,25,26,27,28,29]. However, recent findings show that specific dog breeds’ recorded values must be interpreted based on their normal values [30]. In other words, values obtained from breed-specific echocardiographic studies significantly differ from the general population of healthy dogs of various breeds [9,10,19,24,31,32,33,34]. General population measurements have a wide range and rely on regression analysis and 95% prediction intervals, limiting their clinical usefulness [30]. Breed-specific reference ranges may be more helpful in avoiding misinterpretation of echocardiographic findings [35,36].

In dogs, especially since the advent of M-mode echocardiography, researchers have focused on establishing standard measurements for cardiac structures [31]. Breed-specific, or at least somatotype-specific, normal echocardiographic parameters, such as left ventricular and atrial measurements, are essential for accurate initial cardiac diagnoses and for managing disease progression and severity [34]. As an important amount of information is available in the field of echocardiography, the present review aimed to concentrate the data of the normal reference ranges of LV obtained in M-mode in healthy dogs of both sexes and different ages from all the breeds studied to date. M-mode data on parameters like the left atrium dimension and aortic root dimension were excluded from this review because they were not sufficiently represented within the selected studies. The authors aimed to present the information in a concise, tabular format, benefiting both clinicians and researchers.

## 2. Materials and Methods

**Figure 1:**
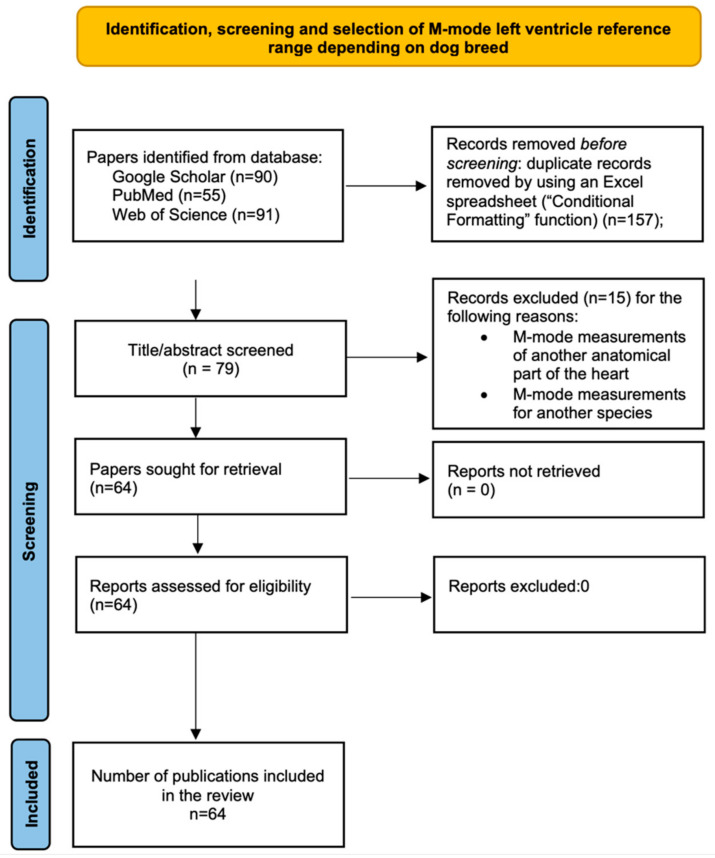
Prisma 2020 flow diagram including searches in Web of Science, PubMed, and Google Scholar databases.

This review was conducted based on reference values obtained from M-mode echocardiography of the left ventricle (LV) for 44 different dog breeds as reported in the scientific literature from three databases. We identified scientific articles through Google Scholar, PubMed, and Web of Science. We conducted systematic searches using the keywords “dog”, “left ventricle”, and “M-mode”, following the protocol in Figure 1, resulting in the consideration of 64 different papers. The data included information on the dogs’ ages, sexes, and sedation status during the echocardiography examination. Additionally, the LV dimensions measured in millimeters were considered, including interventricular septum in diastole (IVSd), left ventricular internal diameter in diastole (LVIDd), left ventricular posterior wall in diastole (LVPWd), interventricular septum in systole (IVSs), left ventricular internal diameter in systole (LVIDs), and left ventricular posterior wall in systole (LVPWs).

All the dogs included in the studies considered for this review met similar inclusion criteria. Before the echocardiographic measurements, the dogs underwent physical examination, and they were found to be free of any abnormal findings. The dogs also had a good body score and normal ECG measurements. Additionally, on transthoracic echocardiography (TTE), none of the enrolled dogs (from the studies used) showed any signs of acquired or congenital heart disease.

## 3. M-Mode Measurements of LV Based on Breed

The following table (Table 1) contains summarized information about the main LV measurements for 44 different dog breeds in an easy-to-read format. For consistency, measuring units were presented in millimeters. Where available, data were provided as intervals rather than mean.

## 4. Results and Discussions

The majority of publications focused on adult dogs, with a few exceptions that included dogs under 12 months of age [30,31,34,38,39,43,48]. Studies on LV parameters in M-mode have revealed notable differences between growing dogs, particularly between puppies and adult dogs [31,38,41,43]. The most significant disparity appears between the first and second month, with cardiac chambers nearly doubling in size. Interestingly, wall thickness does not increase as rapidly, suggesting that ventricular myocardial fibers in postnatal dogs tend to lengthen more than widen. Another possibility is that myocardial density increases with age while extracellular water concentration decreases [31]. In female Beagles, the most rapid heart growth occurs before 7 months of age, consistent with observations in other breeds like the Spanish Mastiff and English Pointer. Corporal growth in female Beagles generally concludes around 9 months, while cardiac growth can be considered complete by 13 months with only slight modifications thereafter [38]. Furthermore, age-associated effects are observed in various parameters for female Beagles, including LVIDd, LVIDs, LVPWd, and LVPWs. The onset of these differences varies by parameter: 7 months for LVIDs; 10 months for LVIDd, LVPWs, Ao, and LA; 13 months for LVPWd and LVEDV, and 17 months for heart rate [38]. In a study involving growing dogs older than 12 months, a positive correlation was observed between age and LVPWs, IVSs, and IVSd in Dogue de Bordeaux. Specifically, mean values of LVPWs and IVSd were significantly influenced by age, with *p* ≤ 0.01 and *p* ≤ 0.05, respectively [41]. In Labrador Retrievers, the mean values of LVPWs for young dogs in age groups 1–2 and 2–3 years were significantly different (*p* ≤ 0.05) from those in the age group 3–5 years [53]. However, no such variations were found in German Shepherd dogs, as there were no statistical differences observed in any of the M-mode echocardiographic parameters with respect to age [25]. In English Bulldogs, left ventricular wall thickness, specifically the left ventricular posterior wall thickness and interventricular septum thickness, increased with age. This phenomenon has been observed previously in both humans and dogs [38,58]. It has been hypothesized that aging results in the gradual loss of myocardial cells, leading to hypertrophy of the neighboring myocytes [42,58]. However, in one of the Chihuahua studies included in the review, age had only a minor impact on LVPWd, which was considered to be of limited clinical significance. This discrepancy is likely because the study primarily included young dogs [39]. In summary, there are significant differences in LV measurements among growing dogs up to 13 months of age, and variations in these measurements in growing adult dogs appear to be breed-dependent.

Two studies exclusively included female dogs, both from the Beagle breed, while no publications focused solely on males. Some studies allowed for the separation of measurements based on sex, and consistent differences were found between genders. For example, in female Beagles, there was a statistically significant difference (*p* < 0.006) in left ventricular wall thickness (LVWT) in systole and diastole [18]. The other study of Beagle could not confirm this as it included females only [38]. One German Shepherd study showed a significant relationship between gender and left ventricular posterior wall thickness in systole and diastole [25]. The other two studies [2,26] could not confirm this difference as the authors did not present different information for males and females. Male Kangal dogs had higher left ventricular posterior wall thickness than females [50]. Significant differences in M-mode left ventricular dimensions (MMLVD) were observed between male and female Great Danes from the United Kingdom [45]; LVIDd (*p* = 0.001) and LVIDs (*p* = 0.011) were significantly lower in female dogs. Similarly, male Dachshunds had larger diastolic and systolic left ventricular diameter than females [30], while adult Cavalier King Charles Spaniels showed a significant but negligible effect of gender [15]. There is a noticeable distinction in values between males and females across various breeds. As shown for Beagle and German Shepard, not all studies on the same breed could verify this since some focused exclusively on one gender or did not account for gender differences.

Some studies conducted measurements on dogs under sedation [33,38,46]. Acepromazine was used at different doses and administration methods, in combination with pethidine or morphine. Sedation status during the examination is clinically relevant, as some anesthetic drugs can interfere with cardiac contractility and impact heart rate, rhythm, preload, afterload, and myocardial contractile and relaxation properties [59,60,61]. Careful consideration of sedation effects is essential for accurate echocardiographic evaluation.

Reference values for eleven breeds (Beagle, German Shepherd, Boxer, Golden Retriever, Whippet, Greyhound, Great Dane, Irish Wolfhound, Labrador Retriever, Dachshunds, and Chihuahua) were described in two to four independent publications. Each study was based on a different number of individuals. Some breeds exhibited similar values to previous studies, while others showed slight variations, possibly due to factors like somatotype and physical activity levels [2,31,44,48].

Mean values for cardiac measurements were shown to be consistent between several studies on different breeds. For example, the mean values for ISTd, ISTs, LVWTd, and LVWTs in Dobermann Pinschers, Malinois, and Dutch Shepherds were similar to those reported in previous studies [17,43,62,63,64]. However, Labrador Retrievers and German Shepherds exhibited slightly higher mean values than Dobermann Pinschers, Malinois, and Dutch Shepherds, possibly due to variations in somatotype [2]. In small breeds, LVIDdN values significantly vary; for instance, Miniature Poodles had a reported LVIDdN of 1.6 ± 0.4, while Toy Poodles, with their smaller size (<5.0 kg), displayed a lower LVIDdN value of 1.342 ± 0.1164 [14,19]. The structure of the upper airway system can influence certain cardiac measurements. However, no significant differences in LV M-mode measurements were found between BOAS+ and BOAS- groups. Similarly, when examining BOAS Grades, no significant differences in echocardiographic measurements between grades were detected [56]. Additionally, the median IVSd and IVSs of pugs in the study of Wiegel et al. [56] were found to be significantly thicker compared with previous studies by Cornell et al. [9] and Esser et al. [21]. It is worth mentioning that these significant deviations in reference intervals persisted despite normalizing pug measurements with the equivalent allometric equations from each respective interbreed study [56].

Recent publications show increased interest in values for small breed dogs, along with Labrador Retrievers, Golden Retrievers, and German Shepherds. Age and body weight are significant factors contributing to value variations. Visser (2019) [10] and Cornell (2004) [9] propose formulas for normalizing values based on dog weight, with the former recognized by ACVIM [11]. Body weight variations in adult dogs of the same breed and gender are low and were not relevant in this review.

To ensure accurate interpretation of echocardiographic examinations in dogs, it is crucial to consider breed-specific reference values. Failing to do so can lead to misinterpretations, falsely indicating heart enlargement or altered activity when compared with general reference ranges, potentially resulting in misdiagnosis and inappropriate treatment [22,29,35,42,54]. The coefficients of variation for all echocardiographically measured parameters can range from 5.03% to 46.43%, with the majority falling below 20% [65]. Notably, the intraobserver category is considered to exhibit the least variation, indicating the best reproducibility, particularly for M-mode and left ventricular volumetric data. Specifically, the coefficient of variation values for left ventricular M-mode measurements is considered reasonably low, suggesting that a change exceeding 10–18% is likely to be significant [65]. Therefore, establishing clinically relevant reference intervals for echocardiographic measurements poses challenges. Combining dogs of all breeds to estimate an overall regression line may lead to overly broad and clinically irrelevant reference ranges. Certain breeds may be misrepresented and misinterpreted by such generalized lines. The sample size used for establishing these ranges is also important, as it impacts the width of the reference range [19]. Table 1 also highlights a significant limitation in the available data for M-mode measurements: the study population size. Most studies have a relatively small sample size that may not represent the entire population. Furthermore, there are notable disparities in the sample sizes across some studies. Although statistical comparisons can be applied to average values or intervals, achieving statistical significance alone may not ensure biological significance. Furthermore, interpreting results from such comparisons can be highly speculative, particularly due to the presence of confounding factors. Therefore, readers should give preference to studies with larger sample sizes when evaluating data.

## 5. Conclusions

Echocardiography remains the primary tool for veterinarians to evaluate the heart. The reference ranges for left ventricle measurements obtained in M-mode have been a subject of controversy and significant interest in veterinary cardiology. A current trend among researchers is to standardize these values according to breed.

Established echocardiographic criteria and applicable reference intervals are crucial for accurate cardiac screening and interpretation. While echocardiographic values for the general canine population have been published, these multibreed prediction intervals are influenced by breed and somatotype. Therefore, the use of breed-specific echocardiographic reference intervals is a more suitable approach for assessing cardiac structure and function.

The results of this review may be useful in the echocardiographic evaluation of cardiac diseases. This could represent a valid option along the values proposed before by Boon (2011) [1], Cornell (2004) [9], Visser (2019) [10], and Esser (2020) [21]. Moreover, the data compiled provide valuable information on the LV dimensions in different dog breeds and will contribute to a better understanding of normal cardiac measurements for various breeds.

## Figures and Tables

**Table 1:** M-mode measurements of LV based on breed.

Breed	No. of Cases	Age	Sex	Sedation	IVSd mm	LVIDd mm	LVPWd mm	IVSs mm	LVIDs mm	LVPWs mm	Study/ Reference
Afghan	20	2–7 years	M and F	N-S	8.0–12.0	33.0–52.0	7.0–11.0	8.0–18.0	20.0–37.0	9.0–18.0	[19]
American Staffordshire Terrier	57	1–10 years	M and F	N-S	5.9–14.3	34.4–51.2	6.2–12.1	8.1–21.6	17.6–36.9	9.3–19.1	[37]
Beagle	25	28 weeks	M	N-S	5–11	18–33	6–13	6–12	8–27	7–17	[18]
	25		F		6–8	21–32	6–12	7–12	9–23	9–13	
Beagle	6	4 months	F	S	7.0 ± 1.3	21.2 ± 1.6	6.4 ± 1.4	10.9 ± 1.9	12.2 ± 1.8	10.4 ± 1.5	[38]
	6	7 months			7.0 ± 1.1	25.5 ± 3.7	8.5 ± 1.7	11.0 ± 2.2	15.6 ± 2.0	11.5 ± 1.1	
	6	10 months			8.5 ± 1.7	25.4 ± 2.0	8.8 ± 1.6	11.6 ± 1.9	15.7 ± 1.8	12.8 ± 1.9	
	6	13 months			7.4 ± 1.9	27.6 ± 3.8	8.2 ± 1.6	12.2 ± 2.1	14.8 ± 2.4	13.3 ± 1.2	
	6	17 months			8.7 ± 0.6	26.9 ± 4.9	9.2 ± 1.6	13.5 ± 1.9	16.1 ± 1.6	13.4 ± 1.5	
	6	21 months			9.1 ± 1.4	27.6 ± 2.4	8.5 ± 0.7	13.5 ± 2.1	16.0 ± 3.1	12.5 ± 1.3	
Border Collie	20	2–12 years	M and F	N-S	7.02–12.66	24.27–41.35	4.94–15.17	8.19–13.60	17.89–31.55	8.06–14.69	[29]
Boxer	81	2.1–11 years	M and F	N-S	8.3–16.1	29.0–48.0	9.0–15.5	8.1–24.6	16.7–33.0	12.2–21.6	[28]
Boxer	37	1–14.5 years	M	N-S	-	40.8 ± 3.0	-	-	28.1 ± 3.9	-	[7]
	48	1–10.8 years	F	N-S	-	38.0 ± 3.2	-	-	26.1 ± 3.0	-	
Cavalier King Charles Spaniel	62	≥12 months	M	N-S	4.8–8.8	23.3–41.1	5.2–8.5	6.9–14.1	13.1–24.6	8.2–14.0	[15]
	72		F		4.8–8.2	21.4–34.7	4.9–8.4	7.0–14.0	10.9–21.0	7.8–14.8	
Chihuahua	25	-	M and F	-	5.2–6.5	15.83–18.53	5.1–6.1	6.95–8.22	9.01–11.52	6.6–8.12	[14]
Chihuahua	47	10 months–7 years	M and F	N-S	2.9–10.00	13.6–23.9	3.0–6.6	-	6.5–13.4		[39]
Dachshund	40	1–7 years	M and F	-	4.6–7.8	21.6–34.5	5.2–8.6	6.7–10.8	11.1–21.1	7.2–12.0	[40]
Dachshund	9	9 months-16 years	M	N-S	5.2–9.6	24.7–34.7	4.3–8.6	7.3–14.9	15.5–22.1	6.9–12.8	[30]
	32		F	N-S	4.5–10.9	17.6–30.0	3.9–11.4	6.4–14.1	7.2–21.0	6.4–14.9	
Dobermann Pinscher	8	3–5 years	-	-	8.3 ± 1.2	41.6 ± 3.4	7.7 ± 1.2	11.8 ± 1.0	28.6 ± 2.5	12.2 ± 1.0	[2]
Dogue de Bordeaux	7 M and 24 F	12–22 months	M	N-S	-	-	12.1–12.3	-	-	-	[41]
			F	N-S	-	-	10.81–11.05	-	-	-	
		22–36 months	M	N-S	-	-	12.56–12.7	-	-	-	
			F	N-S	-	-	11.1–11.38	-	-	-	
		36–50 months	M	N-S	-	-	12.89–13	-	-	-	
			F	N-S	-	-	11.62–11.7	-	-	-	
		50–85 months	M	N-S	-	-	13–13.2	-	-	-	
			F	N-S	-	-	11.82–12.54	-	-	-	
		12–22 months	M and F	N-S	10.14–12.49	-	-	12.41–16.38	-	14.32–17.61	
		22–36 months			11.13–12.5	-	-	13.97–16.4	-	15.69–7.6	
		36–50 months			11.71–13.1	-	-	14.91–17.1	-	16.51–18.27	
		50–85 months			11.8–13.06	-	-	15.1–16.97	-	16.62–18.37	
Dutch Shepherd	5	3–5 years	-	-	10.1 ± 0.9	38.9 ± 8.8	9.8 ± 1.3	13.8 ± 1.5	24.8 ± 5.7	13.0 ± 1.7	[2]
English Bull Terrier	14	9–30 months	M and F	N-S	6–14	32–44	8–12	9–17	kg (93 + 5)–kg (199 + 5)	10–14	[34]
English Bulldog	50	1–14 years	M and F	N-S	6.1–15	31.2–45.5	7–14.7	9–18.2	18.8–29	10.5–20	[42]
English Pointer	16	1 week	M and F	N-S	2.0 ± 0.2	9.0 ± 0.7	2.1 ± 0.3	3.5 ± 0.4	5.1 ± 0.9	4.0 ± 0.5	[43]
	16	2 weeks			2.2 ± 0.5	11.9 ± 1.8	2.4 ± 0.4	4.1 ± 0.5	7.1 ± 1.1	4.5 ± 0.5	
	16	4 weeks			3.2 ± 0.6	14.5 ± 1.7	3.0 ± 0.3	4.8 ± 0.5	8.9 ± 1.5	5.0 ± 0.6	
	16	8 weeks			4.0 ± 0.6	20.3 ± 1.8	4.0 ± 0.6	6.4 ± 0.8	12.2 ± 1.6	6.9 ± 0.7	
	16	3 months			4.8 ± 0.7	26.1 ± 2.0	4.7 ± 0.8	7.7 ± 0.6	16.6 ± 1.6	8.3 ± 0.8	
	16	6 months			6.5 ± 0.8	35.8 ± 2.2	6.4 ± 0.7	9.9 ± 0.8	22.8 ± 2.2	10.6 ± 1.1	
	16	9 months			7.0 ± 1.0	38.1 ± 2.2	7.0 ± 0.6	10.7 ± 1.3	24.5 ± 1.8	11.5 ± 1.2	
	16	12 months			6.9 ± 1.1	39.2 ± 2.4	7.1 ± 0.7	10.6 ± 1.0	25.3 ± 2.4	11.5 ± 1.3	
English Setter	100	19–118 months	M and F	N-S	6.6–12.8	32.7–55.6	6.9–12	7.1–16	21.6–40.5	8.8–15.6	[36]
Flat-Coated Retriever	16	1–11 years	M and F	N-S	8.59 ± 0.93	40.70 ± 2.93	6.80–9.60	11.82 ± 1.37	28.42 ± 2.34	9.80–13.00	[44]
French Bulldog	42	1–10 years	M and F	N-S	7.95 ± 0.99	33.50 ± 4.12	6.20–7.65	11.18 ± 1.69	21.23 ± 3.50	10.92 ± 1.41	[35]
German Shepherd	30	-	M	N-S	9.69 ± 1.528	50.70 ± 4.968	9.91 ± 1.293	13.90 ± 1.720	34.22 ± 3.560	13.94 ± 1.183	[25]
	20	-	F	N-S	9.80 ± 1.355	49.26 ± 4.405	9.11 ± 1.145	14.42 ± 1.525	34.31 ± 3.157	13.22 ± 1.046	
	-	1–2 years	-	N-S	9.93 ± 1.357	49.48 ± 4.416	9.73 ± 1.149	14.37 ± 1.528	33.76 ± 3.165	13.54 ± 1.051	
	-	3 years	-	N-S	10.04 ± 1.321	50.01 ± 4.295	9.34 ± 1.119	14.30 ± 1.487	34.01 ± 3.079	13.40 ± 1.022	
	-	4 years	-	N-S	9.27 ± 1.340	49.99 ± 4.361	9.70 ± 1.134	13.92 ± 1.509	33.79 ± 3.128	13.80 ± 1.038	
	-	>5 years–8 years	-	N-S	9.74 ± 1.442	50.43 ± 4.697	9.27 ± 1.222	14.04 ± 1.625	35.51 ± 3.364	13.58 ± 1.116	
German Shepherd	60	1–5 years	M and F	-	9.6 ± 0.9	41.7 ± 5.0	8.8 ± 1.1	14 ± 0.9	31 ± 5.1	13 ± 1.2	[26]
German Shepherd	10	3–5 years	-	-	10.7 ± 1.7	41.1 ± 4.5	9.3 ± 1.0	14.4 ± 1.5	26.4 ± 4.8	12.7 ± 1.5	[2]
Golden Retriever	20	2–7 years	M and F	N-S	8.0–13.0	37.0–51.0	8.0–12.0	10.0–17.0	18.0–35.0	10.0–19.0	[19]
Golden Retriever	16	1–11 years	M and F	N-S	10.06 ± 1.17	39.89 ± 3.43	8.00–15.33	14.21 ± 1.45	26.67 ± 3.15	11.80–19.40	[44]
Great Dane	15	1–6 years	M and F	N-S	12–16	44–59	10–16	14–19	34–45	11–19	[32]
Great Dane	14	48–143 months	M	N-S	-	53.6	-	-	38.6	-	[45]
	26		F	N-S	-	49.5	-	-	35.6	-	
Greyhound	16	1–4 years	M and F	S	9–14	36–49	10–15	11–16	27–37	13–18	[46]
	16			N-S	8–14	40–49	9–14	10–17	29–38	12–18	
Greyhound	11	5.5 ± 2.5 years	M and F	N-S	10–16	40–50	8–13		28–36		[47]
Greyhound	20	18 months–9 years	M and F	S	11.9	42.7	12.9				[33]
Hungarian Greyhound	22	1–11 years	M and F	N-S	8.6–15.5	37.6–50.4	9.8–15.6	10.1–19.5	21.7–33.3	12.9–19.3	[48]
Hungarian Vizsla	45	6 months–10 years	M and F	N-S	7.1–14.0	30.2–52.3	8.4–15.5	8.8–16.6	18.4–36.4	10.4–20.2	[48]
Indian Spitz	12	3–5 years	M	N-S	6–10	31–47	7–10	8–17	19–29	10–15	[49]
	12		F	N-S	7–9	29–42	6–10	13–17	19–29	10–14	
Indonesian Mongrel	4	2–5 years	M	-	6.15–6.57	26.17–30.61	6.37–9.29	8.63–9.57	13.67–17.55	8.93–12.07	[5]
	5		F	-	5.76–6.54	22.32–30.24	6.69–7.87	8.08–8.78	13.45–20.29	10.89–11.35	
Irish Wolfhound	20	1–9 years	M and F	N-S	9–14.5	46–59	9–13	11–17	33–45	11–17	[32]
Irish Wolfhound	262	12 months–8.5 years	M and F	N-S	5.5–13.5	42.7–65.5	6.6–13.8	8.1–19	25.4–41.5	9.7–21.3	[24]
Italian Greyhound	20	18 months–7 years	M and F	S	6.4	22.2	7.1	-	-	-	[33]
Kangal	25	2–6 years	M		10.6 ± 1.28	55.8 ± 5.04	11.0 ± 1.52	15.3 ± 1.43	39.9 ± 4.29	16.2 ± 1.34	[50]
	25		F		10.1 ± 0.96	55.0 ± 4.17	9.4 ± 0.88	14.9 ± 1.19	38.9 ± 3.61	14.2 ± 1.39	
Labrador Retriever	-	-	-	-	5.6–13.5	27.00–45.30	6.20–11.30	9.10–16.60	14.50–36.80	8.10–20.9	[51]
Labrador Retriever	12	16 months–4 years	M	N-S	5.6–12.5	29.4–45.3	6.8–11.3	8.1–20.8	14.5–36.8	9.4–14.7	[52]
	12	-	F	N-S	6.0–13.5	30.4–42.6	6.2–10.3	10.2–15.3	21.0–30.8	9.1–14.6	
Labrador Retriever	18	-	M	N-S	11.1 ± 0.5	39.3 ± 0.9	8.8 ± 0.3	13.5 ± 0.7	27.5 ± 0.8	11.7 ± 0.4	[53]
	13	-	F	N-S	11.1 ± 0.6	36.5 ± 1.0	8.7 ± 0.4	13.2 ± 0.8	24.8 ± 1.0	11.8 ± 0.5	
	6	1–2 years	-	N-S	10.8 ± 0.8	37.6 ± 1.5	8.1 ± 0.5	12.6 ± 1.2	27.6 ± 1.4	10.3 ± 0.7	
	10	2–3 years	-	N-S	9.9 ± 0.6	37.9 ± 1.2	8.6 ± 0.4	12.6 ± 0.9	26.8 ± 1.1	11.0 ± 0.6	
	8	3–5 years	-	N-S	12.9 ± 0.6	39.7 ± 1.1	8.8 ± 0.4	15.9 ± 0.9	25.6 ± 1.0	13.4 ± 0.5	
	7	>5 years	-	N-S	10.8 ± 0.7	36.2 ± 1.4	9.4 ± 0.5	12.3 ± 1.1	24.5 ± 1.3	12.4 ± 0.6	
Labrador Retriever	14	1–12 years	M and F	N-S	9.60 ± 1.95	40.61 ± 2.91	7.00–14.00	13.91 ± 2.52	27.93 ± 2.49	10.00–21.50	[44]
Labrador Retriever	13	3–5 years	-	-	9.6 ± 1.2	39.7 ± 3.0	8.5 ± 1.1	13.6 ± 1.6	25.6 ± 3.4	13.1 ± 1.3	[2]
Malinois Belgian Shepherd	12	3–5 years	-	-	10.1 ± 0.9	38.9 ± 8.8	9.8 ± 1.3	13.8 ± 1.5	24.8 ± 5.7	13.0 ± 1.7	[2]
Maltese dog	23	2–6 years	M and F	N-S	3.70–6.50	15.80–29.40	3.70–6.20	5.50–10.10	8.30–19.00	5.60–9.40	[54]
Miniature Poodle	20	2–7 years	M and F	N-S	4.0–6.0	16.0–28.0	4.0–6.0	6.0–10.0	8.0–16.0	6.0–10.0	[19]
Mudi	28	1–12 years	M and F	N-S	6.6–10.4	28.4–41.0	7.0–11.3	7.2–12.8	17.7–27.4	8.2–15.6	[48]
New Foundland	27	1–11 years	M and F	N-S	7–15	44–60	8–13	11–20	29–44	11–16	[32]
Nigerian local dogs	20	>1 year	-	N-S	9–18	15–27	5–13	14–23	6–14	9–21	[55]
North American Saluki	83	>12 months	M and F	N-S	7.00–14.00	36.67–55.35	7.47–11.51	10.00–18.00	25.00–42.58	10.00–16.77	[20]
Papillon	4	-	M and F	-	6.79–7.63	17.10–19.85	6.92–7.7	7.88–8.97	10.67–10.95	9.28–9.45	[14]
Pug BOAS -	21	Over 24 months	M and F	N-S	5.7–10.9	21.1–31.0	6.6–10.2	8.2–12.5	10.2–22.4	8.9–14.1	[56]
Pug BOAS +	21		M and F	N-S	5.5–12.8	19.4–31.9	6.6–9.4	8.2–14.8	14.6–24.3	4.4–13.4	
Saluki	110	≥12 months	M and F	N-S	7.7–13.8	33.0–47.3	8.4–12.9	8.9–15.7	23.2–39.8	10.1–17.1	[57]
Spanish Mastiff	13	1 month	-	N-S	4.92 ± 0.15	16.60 ± 0.27	4.73 ± 0.16	7.81 ± 0.19	8.65 ± 0.29	7.61 ± 0.21	[31]
	19	2 months	-	N-S	5.97 ± 0.15	24.82 ± 0.71	5.76 ± 0.14	9.41 ± 0.24	15 ± 0.37	9.24 ± 0.20	
	20	3 months	-	N-S	7.04 ± 0.22	28.29 ± 0.73	7.02 ± 0.25	10.88 ± 0.38	18.67 ± 0.58	10.69 ± 0.39	
	10	4 months	-	N-S	7.20 ± 0.26	35.50 ± 0.60	6.60 ± 0.34	11.30 ± 0.21	22.70 ± 0.57	9.97 ± 0.37	
	11	5 months	-	N-S	7.86 ± 0.41	36.77 ± 0.80	7.09 ± 0.36	12.09 ± 0.51	24.84 ± 0.38	11.23 ± 0.54	
	10	6 months	-	N-S	8.15 ± 0.21	41.95 ± 1.20	7.60 ± 0.29	12.80 ± 0.33	27.55 ± 0.86	11.70 ± 0.45	
	10	7 months	-	N-S	8.35 ± 0.25	42.75 ± 0.53	7.95 ± 0.30	13.05 ± 0.37	27.58 ± 0.55	12.25 ± 0.44	
	10	8 months	-	N-S	8.80 ± 0.17	44.01 ± 1.25	8.25 ± 0.17	13.85 ± 0.33	28.26 ± 1.01	13 ± 0.22	
	10	9 months	-	N-S	9.35 ± 0.36	44.61 ± 1.82	9.05 ± 0.38	14.35 ± 0.54	29.21 ± 0.88	13.80 ± 0.51	
	10	10 months	-	N-S	10.63 ± 0.42	46.05 ± 2.11	10.10 ± 0.42	15.73 ± 0.54	27.51 ± 1.41	14.75 ± 0.50	
	11	11 months	-	N-S	10.76 ± 0.32	47.20 ± 0.88	9.71 ± 0.28	16.25 ± 0.48	29.41 ± 0.83	14.71 ± 0.44	
	10	12 months	-	N-S	11.83 ± 0.47	47.31 ± 1.09	10.80 ± 0.52	17.90 ± 0.61	29.70 ± 1.14	16.30 ± 0.72	
	12	Over 13 months (2–4 years)	-	N-S	9.76 ± 0.42	47.72 ± 1.35	9.71 ± 0.36	15.64 ± 0.50	29.01 ± 1.05	15.17 ± 0.43	
Toy Poodle	40	3–9 years	M and F	-	5.5–6.7	16.49–20.02	5.0–6.4	7.23–9.30	8.99–11.17	7.30–8.87	[14]
Welsh Corgi	20	2–7 years	M and F	N-S	6.0–9.0	28.0–40.0	6.0–10.0	10.0–14.0	12.0–23.0	8.0–13.0	[19]
Whippet	20	18 months–7 years	M and F	S	8,6	35.9	9				[33]
Whippet	105	10–169 months	M and F	N-S	7.1–12.9	25.7–47.5	6.4–11.5	9.0–15.5	17.0–36.1	8.6–17.2	[27]
Whippet	119	≥12 months	M and F	N-S	6.3–12.6	26.8–43.3	6.1–12.1	7.8–14.5	18.8–35.0	9.1–16.0	[57]
Yorkshire	7	3–9 years	M and F	-	4.69–6.10	18.81–19.12	4.95–5.5	7.9–9.1	9.47–11.13	7.32–8.53	[14]

M—male, F—female, N-S—non-sedated, S—sedated, IVSd—interventricular septum in diastole, LVIDd—left ventricular internal diameter in diastole, LVPWd—left ventricular posterior wall in diastole, IVSs—interventricular septum in systole, LVIDs—left ventricular internal diameter in systole, LVPWs—left ventricular posterior wall in systole, BOAS—Brachycephalic Obstructive Airway Syndrome.

## Data Availability

The data presented in this study are available on request from the corresponding author.
